# *In Vivo* therapeutic potential of mesenchymal stem cell-derived extracellular vesicles with optical imaging reporter in tumor mice model

**DOI:** 10.1038/srep30418

**Published:** 2016-07-25

**Authors:** Senthilkumar Kalimuthu, Prakash Gangadaran, Xiu Juan Li, Ji Min Oh, Ho Won Lee, Shin Young Jeong, Sang-Woo Lee, Jaetae Lee, Byeong-Cheol Ahn

**Affiliations:** 1 Department of Nuclear Medicine, Kyungpook National University School of Medicine/Hospital, Daegu 700-721, Republic of Korea; 2 Daegu-Gyeongbuk Medical Innovation Foundation (DGMIF), 80 Cheombok-ro, Dong-gu, Daegu 701-310, Republic of Korea

## Abstract

Mesenchymal stem cells (MSCs) can be used as a therapeutic armor for cancer. Extracellular vesicles (EVs) from MSCs have been evaluated for anticancer effects. *In vivo* targeting of EVs to the tumor is an essential requirement for successful therapy. Therefore, non-invasive methods of monitoring EVs in animal models are crucial for developing EV-based cancer therapies. The present study to develop bioluminescent EVs using *Renilla* luciferase (Rluc)-expressing MSCs. The EVs from MSC/Rluc cells (EV-MSC/Rluc) were visualized in a murine lung cancer model. The anticancer effects of EVs on Lewis lung carcinoma (LLC) and other cancer cells were assessed. EV-MSC/Rluc were visualized *in vivo* in the LLC-efffuc tumor model using optical imaging. The induction of apoptosis was confirmed with Annexin-V and propidium iodide staining. EV-MSC/Rluc and EV-MSCs showed a significant cytotoxic effect against LLC-effluc cells and 4T1; however, no significant effect on CT26, B16F10, TC1 cells. Moreover, EV-MSC/Rluc inhibited LLC tumor growth *in vivo*. EV-MSC/Rluc-mediated LLC tumor inhibitory mechanism revealed the decreased pERK and increased cleaved caspase 3 and cleaved PARP. We successfully developed luminescent EV-MSC/Rluc that have a therapeutic effect on LLC cells in both *in vitro* and *in vivo*. This bioluminescent EV system can be used to optimize EV-based therapy.

Mesenchymal stem cells (MSCs) are multipotent cells that are capable of differentiating into osteoblasts, chondrocytes, and adipocytes, as well as cells of other mesodermal lineages^1^. MSCs can be recruited to sites of inflammation and tissue repair; therefore, the role of MSCs in regenerative medicine and their potential use as tools for gene delivery have been studied extensively^2^. MSCs are also known to migrate to the tumor microenvironment. Much research attention has been given to determining the role of MSCs in cancer progression or treatment; however, the relationship between MSCs and tumor cells remains unclear. Whether MSCs support or inhibit tumor growth has not been determined. MSCs inhibit proliferation and apoptosis of tumor cells^3^. However, some studies have also shown that MSCs can favor tumor growth *in vivo* by promoting tumor vascularization^4^,^5^.

Extracellular vesicles (EVs) are microparticles and bilipid membrane vesicles of endosomal origin^6^ that are secreted from various cell types. They are released into the extracellular space and then enter the vascular system or various biological fluids^7^. EVs are now recognized as nano-sized particles containing proteins, lipids, and genetic material (mRNA and miRNA). These particles are transferred between cells as a method of intercellular communication^8^,^9^,^10^. EVs contain various proteins and RNAs that reflect the contents and functions of the source cells. Currently, investigation of whether EVs can be employed as diagnostic or therapeutic agents is ongoing. In addition to studying their innate therapeutic features, experiments using engineered EVs have also been exploited^6^,^11^,^12^,^13^.

MSC-derived extracellular vesicles (EV-MSC) have unique properties and are characteristic of functional MSCs^14^. EV-MSCs have been suggested as an alternative to MSCs for treating various conditions such as kidney, cardiac, and brain injuries^15^,^16^,^17^. Some *in vitro* studies have demonstrated that human EV-MSCs inhibit cancer cell proliferation in hepatomas, sarcomas, and ovarian and bladder tumor cells^18^,^19^. One study showed that EV-MSCs transport miRNA, proteins, and metabolites to cancer cells^10^. The mesenchymal differentiative phenotype of MSCs involves several mRNAs associated with numerous cell functions regarding the control of transcription, proliferation, and immune cell regulation^20^. Moreover, gene ontology analysis of predicted and validated targets of the miRNAs present in EVs suggests their potential involvement in multi-organ development, survival, differentiation, and regulation of the immune system^21^. In addition, EV-MSCs represent an unappreciated mechanism of intercellular signaling within the tumor microenvironment that may be relevant for the biological effect of MSCs on tumor growth; however, this requires further study^22^. EVs include exosomes and shedding microvesicles (MVs) carry membrane and cytoplasmic constituents of their parental cells, and have been described as a novel mechanism of cell-to-cell communication^23^,^24^. Stem cells are able to release EVs containing specific mRNAs and miRNAs that are transferred to target cells by receptor-mediated mechanisms^10^,^21^,^23^,^25^,^26^. Studies have demonstrated that administering MVs may have some advantages over directly administering MSCs, because MSCs can differentiate into stromal fibroblasts that favor tumor growth; however, MVs can inhibit tumor cell cycle progression without this risk^27^,^28^.

Lung cancer is commonly diagnosed and is a leading cause of cancer-related mortality in both men and women globally^29^. Although multimodal treatments are being used to combat cancer, cancer-related death has increased worldwide and there is an unmet need to find efficacious therapeutic strategies for lung cancer.

Although the use of EVs for cancer therapy has been well-studied for optimization, monitoring the therapy is important for success because targeting the EVs to the tumor is essential *in vivo*. Despite extensive research, few studies have analyzed EV distribution, and thus little is known about their *in vivo* trafficking. Recent studies have labeled EVs for *in vivo* imaging; however, labeling procedures may cause changes in functional aspects of the EVs. When fluorescent dyes like PKH are used for EV lipid labeling, they may not reflect the true half-life of EVs and can be retained in other lipid entities for long periods, thus misguiding the spatiotemporal assessment of EV dynamics especially over long periods. Here, we designed a highly sensitive *Renilla* luciferase (Rluc) reporter system that enables multimodal EV imaging and monitoring *in vivo*. We developed bioluminescent EVs using Rluc-labeled MSCs to visualize the EVs in a mouse lung cancer xenograft model. We also evaluated the therapeutic effect of EVs in this murine lung cancer model using *in vivo* optical imaging.

## Results

### Characterization of MSC/Rluc cells

The Rluc gene was stably transduced into mouse bone marrow-derived MSCs using Rluc-expressing lentiviral particles (Suppl. Figure 1A). The bioluminescent imaging (BLI) signal was higher in MSC/Rluc cells than in parental MSCs, and increased in a cell number-dependent manner (Suppl. Figure 1B; R^2^ = 0.90). The presence of Rluc protein was confirmed by western blot analysis in MSC/Rluc cells, and no band was observed in parental MSCs (Suppl. Figure 1D). Furthermore, Rluc mRNA transcript expression in these cells was analyzed by RT-PCR. Rluc gene expression was detected in MSC/Rluc cells but not in parental MSCs (Suppl. Figure 1C). These results confirmed that Rluc was stably expressed in the MSC/Rluc cells. Next, we confirmed the expression of MSC phenotype markers on these cells using flow cytometry. The MSC/Rluc cells expressed Sca-1, CD29, CD34, and were negative for CD117 (Suppl. Figure 1E). We also analyzed the differentiation potential of MSC and MSC/Rluc cells. MSC and MSC/Rluc cells stained positively for Alizarin Red S after differentiation treatments (Suppl. Figure 1F) for 14 days. The calcium deposits appear as bright orange-red stained areas in the light microscopy images. Both differentiated cultures stained positive (1F (ii) and 1F (iv)) whereas no stain was detected in undifferentiated control cells. These results confirmed that MSC/Rluc cells were capable of osteogenic differentiation, similar to parental MSCs. Furthermore, we compared the proliferation rates of MSC/Rluc cells and parental MSCs. The proliferation rate of MSC/Rluc cells was similar to the rate of parental MSCs after 48 h (Suppl. Figure 1G).

### LLC-effluc activity *in vitro*

LLC-effluc activity, visualized by an IVIS system, increased with increasing cell numbers compared to parental LLC cells (Suppl. Figure 2A). The quantitative data (Suppl. Figure 2B; R^2^ = 0.98) shows a sequential increase in effluc activity. These results confirmed the presence of effluc protein activity in the stable LLC-effluc cells.

### Characterization of EV-MSC/Rluc

**Figure 1:**
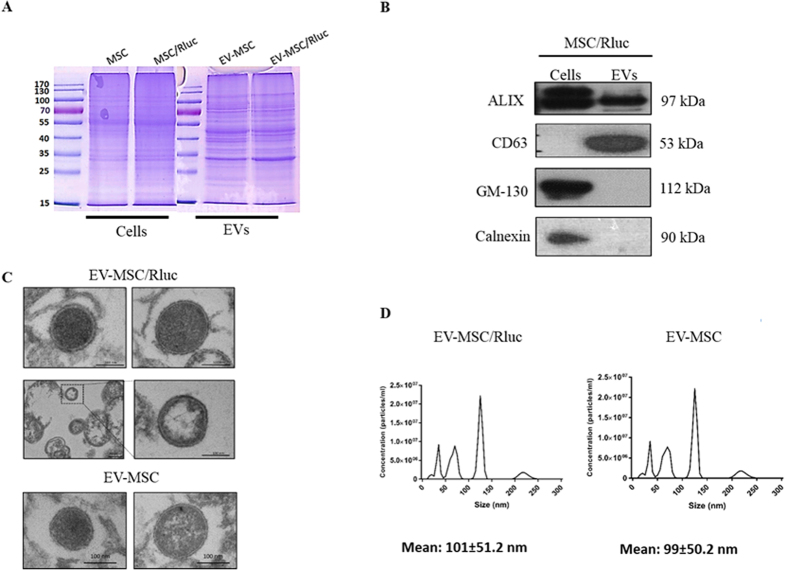
Characterization of EV-MSC and 1 EV-MSC/Rluc. (**A**) Coomassie Brilliant Blue staining. Protein was isolated from naïve MSC and MSC/Rluc cells and their EVs. Equivalent amounts (50 μg) of protein were run on a 10% SDS gel and stained with Coomassie blue. (**B**) Western blotting analysis of EV marker proteins. ALIX and CD63, positive EV marker proteins, were detected in the MSC/Ruc EV. GM-130 (golgi marker) and calnexin (endoplasmic reticulum marker), negative marker proteins for EV, was not detected in MSC/Rluc EVs. (**C**) Analysis of EV-MSC and EV-MSC/Rluc by TEM. Image shows EVs with lipid-bilayer (Scale bars, 100 nm), (**D**) EV-MSC and EV-MSC/Rluc size analyzed by NanoSight. Data are expressed as the mean ± standard deviation (SD) of three independent experiments.

To investigate the effects of MSC and MSC/Rluc derived EVs on tumors, we first isolated EVs from culture supernatants of these cells. The successful purification of MSC/Rluc-derived EVs was examined by western blotting. First, we compared the protein expression profiles of EVs derived from MSC and MSC/Rluc cells with the profiles of their corresponding donor cells. Equal amounts of proteins extracted from MSC and MSC/Rluc derived EVs were separated by 10% SDS-PAGE and stained with Coomassie Blue (Fig. 1A). As shown in Fig. 1B, MSC/Rluc cells and EVs were positive for expression of Alix, a multivesicular body marker. CD63 was detected in EV-MSC/Rluc, whereas GM-130, a Golgi marker, and calnexin, a marker of endoplasmic reticulum, were not detected. The purity of EVs was thus confirmed. Fractions of putative EVs were isolated from conditioned media of MSC and MSC/Rluc cells to investigate the paracrine effect of MSCs via EV release. High-resolution transmission electron micrographs showed that MSC and MSC/Rluc derived EVs exhibited rounded and lipid bilayer structures with a mean size of approximately 100 nm (Fig. 1C). Nanosight analysis was performed to further define the size of these EVs, and the size distribution profile displayed a mean size of around 100 nm for EV-MSC and EV-MSC/Rluc (Fig. 1D). These results demonstrated the successful purification of EV-MSC and EV-MSC/Rluc.

**Figure 2:**
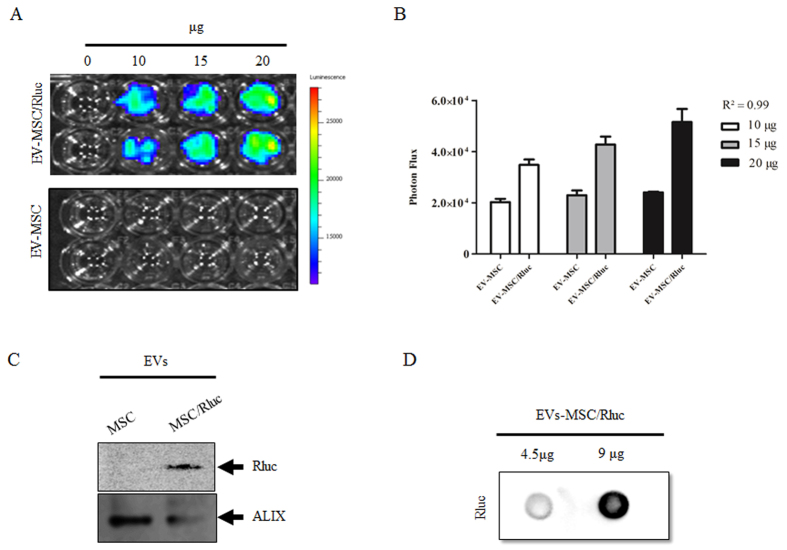
Rluc activity and Rluc protein expression in EV-MSC and EV-MSC/Rluc. (**A**) Representative *BLI* of Renilla luciferase activity of EV-MSC and EV-MSC/Rluc. *BLI* was performed with different concentration of MSC- and MSC/Rluc-derived EVs in a 96 well plate. (**B**) Quantitative Rluc activity of EV-MSC and EV-MSC/Rluc. Data are expressed as the mean ± standard deviation (SD) of three independent experiments. (**C**) Western blot detection of Rluc protein. Rluc protein expression was analyzed in EV-MSC and EV-MSC/Rluc. Alix was immunoprobed as a loading control (**D**) Dot blot detection of Rluc protein in EV-MSC/Rluc.

Next, we confirmed Rluc activity in the EVs using different concentrations of EV-MSC/Rluc and EV-MSC by IVIS upon adding coelenterazine (CTZ). Rluc activity was detected in EV-MSC/Rluc, whereas no bioluminescence was detected in EV-MSC (Fig. 2A). The Rluc activity of EV-MSC/Rluc increased with increasing concentration (10, 15, and 20 μg EVs) compared to EV-MSC upon quantification (Fig. 2B; R^2^ = 0.99). In addition, we confirmed the presence of Rluc proteins in EV-MSC/Rluc by western blotting (Fig. 2C). Furthermore, we also performed a dot blot analysis for Rluc (Fig. 2D). The Rluc protein increased between adding 4.5 μg and 9 μg EV-MSC/Rluc.

### Cellular uptake of EV-MSC/Rluc into LLC-effluc cells

**Figure 3:**
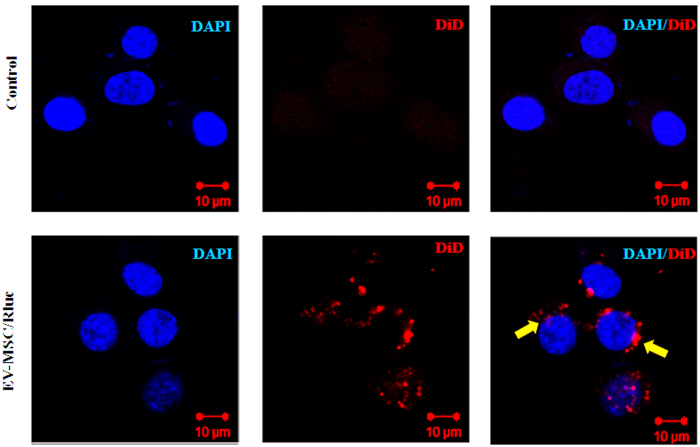
Cellular internalization analysis of MSC/Rluc derived EV into LLC cells by confocal microscopy. LLC cells were incubated with 20 μg of MSC/Rluc-derived EVs that were labelled with DiD for 4 h. Without EV-MSC/Rluc was used as negative control. Scale bar = 10 μm.

To study the internalization of EV-MSC/Rluc by LLC cells, EV-MSC/Rluc were labeled with the fluorescent dye DiD. DiD-labeled EV-MSC/Rluc were incubated with LLC cells for 4 h and cellular uptake of EV-MSC/Rluc was observed by confocal laser microscopy (Fig. 3). We found that DiD-labeled EVs were localized in the LLC cells, indicating that EV-MSC/Rluc were internalized into tumor cells.

### Effect of EV-MSC and EV-MSC/Rluc on LLC luciferase activity and viability of other cancer cell types

**Figure 4:**
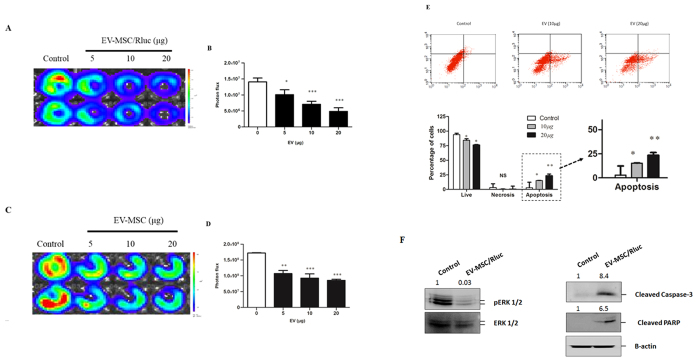
Effect of EV-MSC and EV-MSC/Rluc on LLC-1 effluc activity, and apoptosis mechanism. (**A**,**C**) Representative *BLI* of EV-MSC and MSC/Rluc-EV-treated LLC-effluc activity. The viability of LLC-effluc cells decreased with increasing concentration of EV-MSC and EV-MSC/Rluc. (**B**,**D**) Quantitative effluc activity of LLC-effluc cells. (**E**) Annexin V and PI staining for analyzing LLC cell apoptosis. The percentage of apoptotic cells was increased after treatment with 10 or 20 μg/mL of EV-MSC/Rluc for 24 h. (**F**) Representative western blot analyzing pERK1/2, cleavage of apoptosis markers caspase 3 and PARP levels in EV MSC/Rluc-treated LLC cells. The fold changes were normalized for pERK with total ERK. Cleaved PARP and cleaved caspase 3 normalized with β-actin. Data are expressed as the mean ± standard deviation (SD) of three independent experiments, *p < 0.05, **p < 0.01., ***p < 0.001 (by Student’s *t*-test).

LLC-effluc cells were treated with EV-MSC and EV-MSC/Rluc for 24 h, and then LLC-effluc activity was measured by IVIS imaging (Fig. 4A–D). Various concentrations of EV treatment significantly decreased the viability of LLC-effluc cells. EVs treatment at 5, 10, and 20 μg significantly decreased the LLC-effluc activity (*p* < *0.05*, *0.001*), compared to untreated LLC-effluc cells. The present results confirmed that EVs from parental MSC and Rluc labeled MSC have a similar effect. We also analyzed the therapeutic effect of EV-MSC and EV-MSC/Rluc on other cancer cell types such as melanoma, cervical, breast, and colon cancer, and we found that the breast cancer cell line 4T1 exhibited significantly decreased viability upon EV treatment compared to other types of cancer cells (Suppl. Figure 3).

### Effects of EV-MSC/Rluc on LLC cell apoptosis

Flow cytometric analysis of FITC-Annexin V staining was performed on LLC cells to assess the apoptotic effects of EV treatment (Fig. 4E). LLC cells were treated with EV-MSC/Rluc at 10 or 20 μg/mL for 24 h. After 24 h, cells were incubated with FITC-Annexin V in a buffer containing propidium iodide (PI) and then were subjected to flow cytometric analysis. Untreated cells were primarily FITC-Annexin V- and PI-negative, indicating that they were viable and not undergoing apoptosis. After 24 h treatment with EVs, two populations of cells were observed. Viable cells that were not undergoing apoptosis (FITC-Annexin V and PI negative) constituted 84% and 76% of the total population in cells treated with 10 and 20 μg/mL EV-MSC/Rluc respectively. In contrast, cells undergoing early apoptosis (FITC-Annexin V positive and PI negative) constituted 14.22% and 19.52% of the total cell population treated with 10 or 20 μg/mL EV-MSC/Rluc, respectively, compared to untreated control cells (*p* < *0.05*, *0.001)*. Therefore, the present study confirmed that EV-MSC/Rluc treatment induces apoptosis in LLC cells.

### Mechanism of EV-MSC/Rluc on LLC apoptosis

To determine the EV-MSC/Rluc-mediated cell death mechanism, we examined ERK1/2. Figure 4F depicts the western blot analysis for ERK, phosphorylated ERK, cleaved caspase 3, and cleaved PARP in cells treated with or without EVs. In the present study, EV-MSC/Rluc treatment increased the levels of cleaved caspase 3 (8.4 fold), and PARP (6.5 fold). ERK1/2 is involved in one of the mitogen-activated protein kinase (MAPK) pathways that promote cell cycle progression. In this study, EV-MSC/Rluc decreased pERK1/2 levels (0.03 fold); however, it did not change total ERK levels. The observed decrease in pERK1/2 explains the reduction in LLC, cell viability, and induction of apoptosis.

### *In vivo* visualization of EV-MSC/Rluc and the anti-tumor effect of EVs on LLC tumors

**Figure 5:**
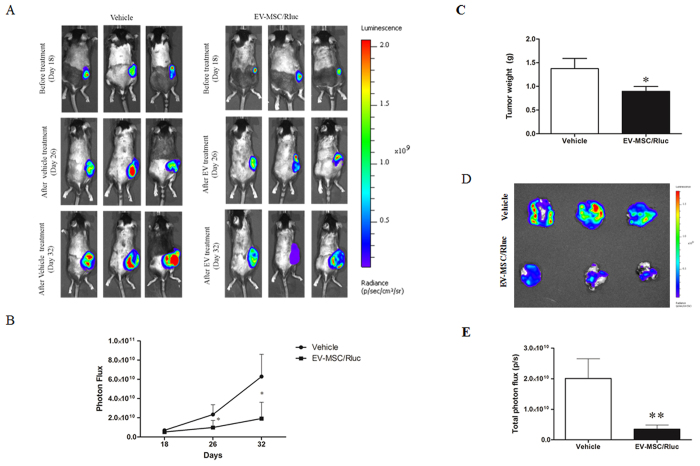
*In vivo* and *ex vivo BLI* to monitor the effect of EV-MSC/Rluc treatment. (**A**) BLI of tumor LLC-effluc activity in mice subcutaneously injected with 1 × 106 LLC-effluc cells and treated with vehicle or EV-MSC/Rluc (50 μg) twice. (**B**) Quantitative BLI of LLC-effluc activity. (**C**) Tumor weight. (**D**) *BLI* imaging of LLC-effluc activity from *ex viv*o tumors (**E**) *Ex vivo* quantitative *BLI* of LLC-effluc activity. Values are expressed as the mean ± standard deviation (SD), *p < 0.05, **p < 0.01 (by Student’s *t*-test).

To determine the effect of EVs on tumor growth *in vivo*, immune-competent (C57BL6) mice were utilized. Intratumor injection of 50 μg EV-MSC/Rluc was performed at days 21 and 26 after xenograft implantation. A schematic diagram of the *in vivo* study is depicted in Suppl. Figure 4. The Rluc activity of mice injected intratumorally with EV-MSC/Rluc is shown in Suppl. Figure 5. Rluc activity of EV-MSC/Rluc was successfully visualized in LLC tumors. The LLC-effluc activity of the tumors upon IVIS imaging is shown in Fig. 5. Five days after the second injection of EV-MSC/Rluc, the LLC-effluc activity was analyzed. The LLC-effuc activity of the EV-MSC/Rluc treated group was decreased compared to the activity of the vehicle-treated group (Fig. 5A,B) on day 26 (*p* < *0.05*) and day 32 (*p* < *0.05*). The tumor weight (Fig. 5C) in the EV-MSC/Rluc-treated group was also decreased significantly compared to the vehicle-treated group (*p* < *0.05*). In addition, the *ex vivo* effluc activity (Fig. 5D,E) of EV-MSC/Rluc treated tumors was also decreased significantly compared to effuc activity in control tumors (*p* < *0.001*).

## Discussion

In the current study, we demonstrated the presence of the Rluc reporter protein in EVs derived from Rluc-expressing MSCs, as well as the visualization of EV-MSC/Rluc in the LLC-xenograft tumor model. We also studied the anticancer effect of EV-MSC/Rluc, and to our knowledge, this is the first study to examine MSC-derived EVs with an Rluc reporter. EVs, or exosomes, are nano-sized vesicles of endocytic origin and are released by multiple cell types to function as mediators of intercellular communication by transferring RNA and proteins^30^,^31^. MSC-derived EVs carry a large cargo load and protect their contents from chemicals or degradative enzymes^32^. Microparticles derived from MSCs could represent a novel therapeutic tool. These vesicles have significant effects on target cells, and this treatment approach has been exploited in many diseases, such as cardiovascular diseases, renal diseases, and cancer^6^,^18^,^32^,^33^.

First, we successfully transduced the Rluc reporter gene into MSCs and isolated EVs from the culture supernatant of these MSC/Rluc cells. We observed that MSC-derived EVs showed unique protein expression patterns as reported by studies with other cells^34^,^35^. The EV purity was confirmed by western blot analysis for CD63, which is a positive marker of EVs^36^. Alix is a cargo protein of the multivesicular body (MVB) that was observed in both cells and EVs. Further, the absence of GM130 and calnexin in EVs showed that they were free from contamination with cell organelles. These results confirmed that the EVs isolated from MSC/Rluc cells were pure. The size and morphology of EVs were examined with TEM, and the mean size of EV-MSC and EV-MSC/Rluc, as confirmed by NanoSight analysis, was about 100nm, which is similar to the findings of previous studies^37^,^38^,^39^.

Further, we confirmed Rluc activity and the presence of Rluc protein in the EV-MSC/Rluc. These results suggest selective distribution of the reporter protein in EVs and support the observations of previous studies with other reporter proteins^40^,^41^. Therefore, these results showed that this novel bioluminescent reporter protein can be used to label EVs.

MSCs can be isolated from different sources, mainly the bone marrow and adipose tissue, and can differentiate into various cell types of the mesodermal lineage. The characteristics of MSCs make them good candidates for therapeutic intervention in regenerative medicine or immunologic disorders^42^. Furthermore, MSCs have the potential for oncologic application as they are known to inhibit tumor progression when administered into established tumors^43^,^44^,^45^. However, MSCs also can have oncogenic potential, as was found for several tumor types, including osteosarcomas, lipomas, and gastric adenocarcinomas^46^,^47^,^48^, strongly suggesting their involvement in tumor development. Therefore, any clinical application of MSCs needs to be evaluated cautiously. In addition, the timing of MSC injection is crucial for therapeutic intervention^22^. Some studies have demonstrated that the co-injection of MSCs with tumor cells promotes tumor growth^49^,^50^. Therefore, administering EVs may have some advantages over administering MSCs. There is growing interest in using EVs as biological delivery vehicles. EVs can be taken up by acceptor cells, and thereby a number of cellular processes can be altered^51^. In the present study, EV-MSC/Rluc were taken up by LLC cells, as confirmed by confocal imaging. Alvarez *et al.* provided the first proof-of concept for biotechnological exploitation of EVs^13^. Christianson *et al.* reported that exosomes mediate intercellular transfer by cell surface heparin proteoglycans, however the mechanism of exosome uptake and function on target cells remains unknown^52^. The internalized exosomes reside in common vesicular structures containing syndecan and glypican, indicating that heparin proteoglycans act as internalizing receptors for exosomes^52^.

There is evidence that EVs do not elicit acute immune rejection; they are non-viable, and thus they do not have tumorigenic potential. MSC-derived EVs can be mass-produced in culture and can be incorporated with multiple therapeutic miRNAs, thus enabling personalized treatment^51^,^53^,^54^. Moreover, MSC-derived EVs have no risk of aneuploidy, teratoma formation, or immune rejection after allogeneic administration *in vivo*^20^,^55^,^56^. Hence, use of MSC-derived EVs for disease treatment is a promising therapeutic strategy. In the present study, exposure to EV-MSC/Rluc resulted in inhibition of LLC tumor growth *in vitro* and *in vivo.* EV-MSC and EV-MSC/Rluc significantly inhibited LLC-effluc activity with increasing concentrations. In addition, we analyzed the effects of EV-MSC and EV-MSC/Rluc on the viability of other cancer cells and found that 4T1 cells were significantly inhibited by EV treatment; however, no significant changes were observed in other cancer cells such as B16F10, TC-1, and CT26. Although MSC-derived EVs are not effective on all types of cancers, they have definitive therapeutic effects on certain types of cancers. Further, induction of LLC cell apoptosis was observed upon EV-MSC/Rluc treatment at doses of 10 and 20 μg/mL. These data indicate that MSC/Rluc derived EVs have the potential to inhibit LLC.

In order to demonstrate the mechanism of cell death induced by EV treatment, we analyzed ERK, pERK, cleaved caspase 3, and cleaved PARP. The most important signaling molecule of the caspase cascade is caspase 3, which is a downstream effector caspase^57^,^58^. Activation of caspase 3 then leads to the cleavage of cellular substrates like PARP (poly (ADP)-ribose polymerase)^59^. In the present study, EV-MSC/Rluc treatment inhibited mitogenic signaling by downregulating pERK1/2 and activated apoptosis signaling by increasing cleaved caspase 3 and cleaved PARP. The EV-MSC/Rluc-mediated induction of apoptosis was also confirmed by flow cytometry with annexin V staining.

The advent of molecular imaging technologies, coupled with the development of cell-based therapies, has brought about a revolution in *in vivo* visualization. Among optical imaging tools, bioluminescence imaging with reporter genes is an indirect or direct labeling technique and is a promising method for visualizing biological targets in small animal models. Bioluminescence is generated by the conversion of chemical energy into visible light by the action of luciferase enzymes on their substrates^60^,^61^,^62^,^63^. In the current study, EV-MSC/Rluc isolated from MSC/Rluc were injected intratumorally and were well visualized with BLI upon addition of CTZ in the LLC xenograft tumor model. We confirmed that the bioluminescent EVs retained Rluc activity in the *in vivo* tumor model.

For EV treatment of tumors *in vivo*, LLC cells labeled with effluc were used and the therapeutic effect of EVs on the tumor xenograft was monitored non-invasively by BL imaging. EV-MSC/Rluc (50 μg) were administered twice with a 5 day interval by direct intratumor injection in the rapidly growing LLC tumors, and PBS was injected as the vehicle control. EVs could inhibit tumor growth and reduce tumor size. Previously, Bruno *et al.* injected MSC-derived MVs weekly in an established tumor and found tumor regression upon MV injection^15^. Our *in vivo* study also observed inhibition of tumor growth by EV-MSC/Rluc injection in LLC tumor xenografts.

In the current study, we visualized both the EVs and xenografts in animals in a non-invasive manner using two different luciferase reporter genes combined with the appropriate substrates and a two-color reporter system. This is an ideal system to visualize the therapeutic agent and the target together in the same animal and can be used to optimize EV therapy and other therapeutic options as well.

Additional studies are still needed to optimize the delivery of EVs to multiple tumors via systemic administration for better clinical translation. Technological improvements for culture and purification of EVs are needed to increase the efficiency of EV isolation and EV safety, in order to determine the feasibility of MSC-derived EV therapy. Optimization of EV therapy can improve the therapeutic effect either when used alone or in combination with other therapeutic modalities.

In the current study, an Rluc reporter system in MSC-derived EVs was successfully utilized and the presence of Rluc protein was confirmed in the EVs. EV-MSC/Rluc were non-invasively visualized in an LLC tumor xenograft model. In addition, the MSC-derived EVs were found to inhibit proliferation of LLC cells *in vitro* and to inhibit progression of LLC tumor xenografts *in vivo*. Thus, our findings suggest that MSC-derived EVs can be effective therapeutic agents against lung cancer.

## Materials and Methods

### Cell culture

Mouse bone marrow-derived MSCs (Invitrogen, Carlsbad, CA, USA) were cultured in DMEM-F12 (HyClone, Logan, UT, USA) supplemented with 10% fetal bovine serum (Hyclone), 1% gentamicin (GIBCO-BRL Life Technologies, Gaithersburg, MD, USA), and 1 × Glutamax (Invitrogen). The mouse lung cancer cell line, Lewis lung carcinoma (LLC), was obtained from the American Type Culture Collection (Manassas, VA, USA). The LLC cell line was cultured in DMEM-High (Hyclone) containing 10% fetal bovine serum and 1% penicillin-streptomycin. All cells were cultured at 37 °C in 5% CO_2_.

### Transduction of the Rluc gene into MSCs

MSCs were transduced with lentiviral particles expressing Rluc under the control of the CMV promoter (Genecopoeia, Rockville, MD, USA) and then cells stably expressing Rluc were selected with 2 μg/mL puromycin (MSC/Rluc cells) for 2 weeks.

### Confirmation of Rluc activity in MSC/Rluc cells

To confirm Rluc activity, MSCs and MSC/Rluc cells at were plated at various densities (1.25 × 10^4^, 2.5 × 10^4^, 5 × 10^4^, 1 × 10^5^, and 2 × 10^5^ cells/well) in a white and clear-bottom 24-well plate in DMEM-F12 medium. After 24 h, the substrate coelenterazine (CTZ) (Perkin Elmer, Waltham, MA, USA) was added to each well, and Rluc activity was determined by BLI (Bioluminesence imaging), using an IVIS Lumina II system (Caliper Life Sciences, Hopkinton, MA USA).

### Characterization of MSC/Rluc cells and phenotype marker analysis

The selected MSC/Rluc cells were characterized for the presence of MSC-specific markers, which were analyzed by RT-PCR and western blotting. MSCs and MSC/Rluc (0.5 × 10^6^ cells) were incubated with 1 μg of phycoerythrin (PE)-conjugated primary antibodies (Sca-1, CD-29, CD117, and CD-34) or isotype-matched control immunoglobulin G (IgG1) (BD, Franklin Lakes, NJ, USA) at 4 °C for 30 min. Samples were analyzed by flow cytometry (BD Accuri C6; BD Biosciences, San Jose, CA, USA).

### Differentiation of MSC and MSC/Rluc cells

To confirm the differentiation of naïve MSCs and Rluc-labeled MSCs, we analyzed their osteogenic potential using a previously described protocol with slight modifications^64^. MSCs and Rluc-labeled MSCs (2 × 10^4^ cells/well) were seeded in 6-well plates and were incubated in osteogenic medium (DMEM-F12, FBS 5%, 50 μg/mL ascorbic acid, 10 mM β-glycerophosphate, 10 nM dexamethasone) for 14 days, with medium changes every 2–3 days. After 14 d, cells were fixed in 10% formalin and stained with 2% Alizarin Red S (Sigma Aldrich).

### CCK-8 cell proliferation assay for naïve MSC and MSC/Rluc cells

An *in vitro* proliferation assay was performed on MSCs and MSC/Rluc cells using a CCK-8 assay kit (Dojindo Laboratories, Kumamoto, Japan). Briefly, MSCs and MSC/Rluc cells (5,000 cells/well) were plated in 96-well plates and were incubated for 48 h at 37 °C, 5% CO_2_. After 48 h, 10 μL of CCK-8 solution was added to each well followed by incubation for 1 h at 37 °C. Absorbance at 450 nm was then measured using a microplate reader.

### Transduction of enhanced firefly luciferase (effluc) gene into LLC cells

LLC cells were cultured in DMEM-High (Hyclone) supplemented with 10% fetal calf serum and 1% antibiotic-antimycotic solution (Gibco), at 37 °C and 5% CO_2_. LLC cells were transduced with effluc-expressing retroviral particles isolated from Phoenix A-effluc cells. The transduced LLC-effluc cells were sorted with magnetic beads bound to a CD90.1 MicroBeads (thy1) antibody (MACS, Miltenyi Biotec, South Korea) for selecting effluc positive cells, and their effluc activity was quantified by IVIS imaging using D-luciferin as a substrate.

### Isolation of EVs

MSC and MSC/Rluc-derived EVs were isolated by an ultracentrifugation method. Briefly, MSCs and MSC/Rluc cells were cultured in the presence of FBS depleted of EVs (by centrifugation at 100,000 × *g* for 18 h at 4 °C). EVs were then purified from the cell-free supernatant according to a previously described protocol with slight modifications^65^. After 2–3 days of culture, the cell supernatants were collected and centrifuged at 300 × *g* for 10 min to remove live cells, then at 1500 × *g* for 15 min to remove cell debris, and finally at 2500 × *g* for 20 min to remove apoptotic bodies. This supernatant was filtered through a 0.45 μm syringe filter and centrifuged at 100,000 × *g* for 60 min at 4 °C to obtain EVs. To remove protein contamination, the EV pellet was washed by resuspension in phosphate buffered saline (PBS) followed by another centrifugation at 100,000 × *g* for 60 min. All centrifugations were performed at 4 °C. The residual pellet was resuspended in 50–100 μL of PBS, stored at −20 °C, and functional assays were performed within a week. Ultracentrifugation was performed (SW28 rotor; Ultra-Clear tube) using the Optima L-100 XP ultracentrifuge (Beckman Coulter, USA). The protein content of EVs was quantified by the bicinchoninic acid method (BCA kit, Pierce, Appleton, WI, USA).

### Western blot analysis and dot blot for Rluc

For western blot analysis, protein samples (30 to 80 μg) were separated using 10% SDS-PAGE and then were transferred to PVDF membranes (Millipore, USA). The blots were incubated with primary antibodies against Rluc (GeneTex, USA), Alix (Abcam), GM-130 (Abcam), calnexin (Abcam), CD63 (Abcam), pERK, ERK (Santa Cruz Biotechnology, Inc., Santa Cruz, CA, USA), cleaved caspase 3 (Cell Signaling Technology, USA), and cleaved PARP (Cell Signaling Technology, USA) followed by incubation with the appropriate HRP-tagged secondary antibody (Santa Cruz Biotechnology, Inc., Santa Cruz, CA, USA). The protein-antibody complexes were visualized using an enhanced chemiluminescence kit (Pierce, USA). The dot blot method for detecting proteins was performed as described previously^66^. Here, Rluc protein was detected from EV-MSC/Rluc in PBS spotted onto nitrocellulose membranes at protein amounts of 4.5 and 9 μg, by a 2-h incubation with Rluc antibody followed by incubation with streptavidin-HRP conjugate and detection by chemiluminescence. *ImageJ* 1.38 software was used to quantify the band intensity (Windows version of NIH Image; http://rsb.info.nih.gov/nih-image/).

### Transmission electron microscopy (TEM)

The MSC and MSC/Rluc EV pellets were obtained by ultracentrifugation and fixed at 4 °C overnight. The fixative contained 2.5% glutaraldehyde in 0.01 M phosphate buffer at pH 7.4 (filtered through 0.22 μm filters) and cells were washed with PBS. EVs were post-fixed in 1% OsO_4_ (Taab Laboratories Equipment Ltd.) for 30 min. EV pellets were washed with distilled water and dehydrated with graded ethanol solutions. EV pellets were negatively stained with 1% uranyl-acetate in 50% ethanol for 30 min and embedded in Taab 812 (Taab), followed by overnight polymerization at 60 °C and ultrasectioning for TEM. Ultrathin sections were examined and images were captured with a HT 7700 transmission electron microscope (Hitachi, Tokyo, Japan), operated at 100 kV.

### Nanoparticle Tracking Analysis

EV-MSC and EV-MSC/Rluc resuspended in PBS were further diluted (1 μl/mL) in distilled water for measuring size distribution using the NanoSight LM10 instrument. This method is based on conventional optical microscopy and uses a laser light source to illuminate nano-scale particles. The EV solutions (1 mL) were introduced into the viewing unit using a disposable syringe. Enhanced with a perfect black background, individual particles appeared as point-scatters moving under Brownian motion. The Nanoparticle Tracking Analysis (NTA) software suite allowed automatic tracking and size determination of the nanoparticles on an individual basis. Results are displayed as a frequency size distribution graph.

### Cellular uptake of EV-MSC/Rluc

The cellular uptake of EVs was analyzed by a modified chemical protocol^34^. EV-MSC/Rluc (20 μg) were incubated with DiD (Invitrogen) for 20 min at room temperature, followed by washing with PBS and ultracentrifugation. The supernatant was removed and pellets were resuspended in 50 μL of PBS. The DiD-labeled MSC/Rluc-EVs were incubated with LLC cells for 4 h at 37 °C under 5% CO_2_. After incubation, cells were washed twice with PBS and fixed in 4% paraformaldehyde for 10 min at room temperature. Samples were washed twice with PBS and mounted on a coverslip using Vecta mounting medium containing DAPI (Vector Laboratories, Burlingame, CA, USA). Cellular uptake of MSC-derived EVs was observed by confocal laser microscopy (Zeiss, LSM 5 exciter, Germany).

### Effect of EV-MSC and EV-MSC/Rluc on LLC cell viability *in vitro*

LLC-effluc cells were seeded at a density of 5,000 cells/well into 96-well plates in 100 μL/well of DMEM-high glucose with 10% FBS. After overnight incubation, the cells were treated with different concentrations of EV-MSC and EV-MSC/Rluc (5, 10, 20 μg) for 24 h and then LLC-effluc activity was assessed by IVIS imaging.

#### Cancer cell viability by CCK assay

The cell viability of CT26, B16F10, TC-1, and 4T1 cancer cell lines was analyzed by CCK-8 assay kit after treatment with EV-MSC and EV-MSC/Rluc. The various cancer cells were seeded in 96-well plates at a density of 5 × 10^3^ cells per well. The EVs were added into the wells and incubated for 24 h. Further, the cells were grown in a CO_2_ incubator at 37 °C and 5% CO_2_. After 24 h, 10 μL of CCK-8 solution was added to each well followed by incubation for 1 h. Then, the absorbance at 450 nm was measured using a microplate reader (Bio-rad). The OD values were plotted.

### Quantification of apoptosis by flow cytometry with Annexin V and PI staining

Apoptosis was assessed using Annexin V, a protein that binds to phosphatidylserine (PS) residues exposed on the surfaces of apoptotic cells. Annexin V-FITC/propidium iodide (PI) double-staining was performed with an Annexin V-FITC Kit (BD Bioscience, San Jose, CA, USA). LLC cells were treated with different concentrations of EVs for 24 h. The cells were then trypsinized, rinsed twice with PBS, and resuspended in 1 × binding buffer. These cells were labeled with 10 μL of FITC-conjugated Annexin V antibody and 10 μL of propidium iodide. The cells were incubated for 20 min in the dark at 37 °C, and then 450 μL of binding buffer was added and the samples were immediately analyzed with a flow cytometer (BD Bioscience). The Annexin V-FITC−/PI− cell population was considered normal, whereas the Annexin V-FITC+/PI− and Annexin V-FITC+/PI+ cell populations were indicative of early and late apoptotic cells, respectively.

### *In vivo* visualization of EV-MSC/Rluc and anti-tumor effects of EVs on LLC tumors

LLC-effluc cells (1 × 10^6^) were subcutaneously implanted into male C57BL/6 mice (Charles River, Jackson Laboratories). Tumors were allowed to grow for 2–3 weeks after implantation, and tumor establishment was assessed by measuring effluc activity using IVIS bioluminescence imaging. Animals with tumors exhibiting near similar photon flux values were used for EV treatment. The treatment protocol used for this study has been described previously, with slight modifications^15^. Mice were randomized into two treatment groups: (1) the control group was injected with vehicle (PBS) alone (n = 3); (2) the EV group received intratumoral injections of EVs (n = 3). The intratumor injection of EV-MSC/Rluc was initiated on day 21 after xenograft implantation. The first treatment was 50 μg of EV-MSC/Rluc in 25 μL of PBS. IVIS bioluminescence imaging was performed to assess the *in vivo* Rluc activity of the injected EV-MSC/Rluc. The second dose on day 26 after xenograft implantation was identical to the dosage given in the first treatment. LLC-effluc activity was monitored on days 26 and 32. Five days after the second EV treatment dose, the mice were sacrificed and tumors were harvested to examine *ex vivo* effluc activity and tumor weight.

### Ethics Statement

All described procedures were reviewed and approved by Kyungpook National University (KNU-2012-43) Animal Care and Use Committee, and performed in accordance with the Guiding Principles for the Care and Use of Laboratory Animals.

### Statistical analysis

All data are expressed as means ± standard deviation (SD). Data from the two experimental groups were analyzed by t-test using GraphPad Prism5 software version 5.01 (GraphPad Software, Inc. USA). A *P value* less than 0.05 was considered statistically significant.

## Additional Information

**How to cite this article**: Kalimuthu, S. *et al.* *In Vivo* therapeutic potential of mesenchymal stem cell-derived extracellular vesicles with optical imaging reporter in tumor mice model. *Sci. Rep.* **6**, 30418; doi: 10.1038/srep30418 (2016).

## Supplementary Material

Supplementary Information

## References

[b1] Uccelli A., Moretta L. & Pistoia V. Mesenchymal stem cells in health and disease. Nat. Rev Immunol. 8, 726–736 (2008). 19172693 10.1038/nri2395

[b2] Bergfeld S. A. & DeClerck Y. A. Bone marrow-derived mesenchymal stem cells and the tumor microenvironment. Cancer. Metast Rev. 29, 249–261 (2010). 10.1007/s10555-010-9222-7 20411303

[b3] Ramasamy R. *et al.* Mesenchymal stem cells inhibit proliferation and apoptosis of tumor cells: impact on *in vivo* tumor growth. Leukemia. 21, 304–310 (2007). 17170725 10.1038/sj.leu.2404489

[b4] Yu J. M., Jun E. S., Bae Y. C. & Jung J. S. Mesenchymal stem cells derived from human adipose tissues favor tumor cell growth *in vivo*. Stem Cells Dev. 17, 463–474 (2008). 18522494 10.1089/scd.2007.0181

[b5] Zhu W. *et al.* Mesenchymal stem cells derived from bone marrow favor tumor cell growth *in vivo*. Exp. Mol Pathol. 80, 267–274 (2006). 16214129 10.1016/j.yexmp.2005.07.004

[b6] Lai R. C., Chen T. S. & Lim S. K. Mesenchymal stem cell exosome: a novel stem cell-based therapy for cardiovascular disease. Regen Med. 6, 481–492 (2011). 21749206 10.2217/rme.11.35

[b7] Zhang H.-G. *et al.* Exosomes and immune surveillance of neoplastic lesions: a review. Biotech Histochem. 87, 161–168 (2012). 22216980 10.3109/10520291003659042 PMC3445025

[b8] Wiklander O. P. *et al.* Extracellular vesicle *in vivo* biodistribution is determined by cell source, route of administration and targeting. J. Extracell. Vesicles. 4, 26316, doi: 26310.23402/jev.v26314.26316 (2015). 25899407 10.3402/jev.v4.26316 PMC4405624

[b9] Andaloussi S. E., Mäger I., Breakefield X. O. & Wood M. J. Extracellular vesicles: biology and emerging therapeutic opportunities. Nat. Rev. Drug Discov. 12, 347–357 (2013). 23584393 10.1038/nrd3978

[b10] Valadi H. *et al.* Exosome-mediated transfer of mRNAs and microRNAs is a novel mechanism of genetic exchange between cells. Nat. Cell Biol. 9, 654–659 (2007). 17486113 10.1038/ncb1596

[b11] Gatti S. *et al.* Microvesicles derived from human adult mesenchymal stem cells protect against ischaemia–reperfusion-induced acute and chronic kidney injury. Nephrol. Dial. Transp., 1474–1483 (2011). 10.1093/ndt/gfr015 21324974

[b12] Zitvogel L. *et al.* Eradication of established murine tumors using a novel cell-free vaccine: dendritic cell derived exosomes. Nat. Med. 4, 594–600 (1998). 9585234 10.1038/nm0598-594

[b13] Alvarez-Erviti L. *et al.* Delivery of siRNA to the mouse brain by systemic injection of targeted exosomes. Nat. Biotechnol. 29, 341–345 (2011). 21423189 10.1038/nbt.1807

[b14] Baglio S. R. *et al.* Human bone marrow-and adipose-mesenchymal stem cells secrete exosomes enriched in distinctive miRNA and tRNA species. Stem. Cell Res. Ther. 6, 127, doi: 110.1186/s13287-13015-10116-z (2015). 26129847 10.1186/s13287-015-0116-z PMC4529699

[b15] Bruno S. & Camussi G. Role of mesenchymal stem cell-derived microvesicles in tissue repair. Pediatr. Nephrol. 28, 2249–2254 (2013). 23386109 10.1007/s00467-013-2413-z

[b16] Bruno S., Collino F., Iavello A. & Camussi G. Effects of mesenchymal stromal cell-derived extracellular vesicles on tumor growth. Front. Immunol. 5, 382, doi: 310.3389/fimmu.2014.00382 (2014). 25157253 10.3389/fimmu.2014.00382 PMC4127796

[b17] Katsuda T., Kosaka N., Takeshita F. & Ochiya T. The therapeutic potential of mesenchymal stem cell‐derived extracellular vesicles. Proteomics. 13, 1637–1653 (2013). 23335344 10.1002/pmic.201200373

[b18] Bruno S. *et al.* Microvesicles derived from human bone marrow mesenchymal stem cells inhibit tumor growth. Stem Cells. Dev. 22, 758–771 (2012). 23034046 10.1089/scd.2012.0304

[b19] Wu S., Ju G.-Q., Du T., Zhu Y.-J. & Liu G.-H. Microvesicles derived from human umbilical cord Wharton’s jelly mesenchymal stem cells attenuate bladder tumor cell growth *in vitro* and *in vivo*. PloS one. 8, e61366, doi: 61310.61371/journal.pone.0061366 (2013). 23593475 10.1371/journal.pone.0061366 PMC3625149

[b20] Bruno S. *et al.* Mesenchymal stem cell-derived microvesicles protect against acute tubular injury. J. Am. Soc. Nephrol. 20, 1053–1067 (2009). 19389847 10.1681/ASN.2008070798 PMC2676194

[b21] Collino F. *et al.* Microvesicles derived from adult human bone marrow and tissue specific mesenchymal stem cells shuttle selected pattern of miRNAs. PloS one. 5, e11803, doi: 11810.11371/journal.pone.0011803 (2010). 20668554 10.1371/journal.pone.0011803 PMC2910725

[b22] Klopp A. H., Gupta A., Spaeth E., Andreeff M. & Marini F. Concise review: dissecting a discrepancy in the literature: do mesenchymal stem cells support or suppress tumor growth? Stem Cells. 29, 11–19 (2011). 21280155 10.1002/stem.559 PMC3059412

[b23] Ratajczak J., Wysoczynski M., Hayek F., Janowska-Wieczorek A. & Ratajczak M. Membrane-derived microvesicles: important and underappreciated mediators of cell-to-cell communication. Leukemia. 20, 1487–1495 (2006). 16791265 10.1038/sj.leu.2404296

[b24] Schorey J. S. & Bhatnagar S. Exosome function: from tumor immunology to pathogen biology. Traffic. 9, 871–881 (2008). 18331451 10.1111/j.1600-0854.2008.00734.x PMC3636814

[b25] Deregibus M. C. *et al.* Endothelial progenitor cell–derived microvesicles activate an angiogenic program in endothelial cells by a horizontal transfer of mRNA. Blood. 110, 2440–2448 (2007). 17536014 10.1182/blood-2007-03-078709

[b26] Yuan A. *et al.* Transfer of microRNAs by embryonic stem cell microvesicles. PloS one. 4, e4722, doi: 4710.1371/journal.pone.0004722 (2009). 19266099 10.1371/journal.pone.0004722 PMC2648987

[b27] Spaeth E. L. *et al.* Mesenchymal stem cell transition to tumor-associated fibroblasts contributes to fibrovascular network expansion and tumor progression. PloS one. 4, e4992, doi: 4910.1371/journal.pone.0004992. (2009). 19352430 10.1371/journal.pone.0004992 PMC2661372

[b28] Roorda B. D., ter Elst A., Kamps W. A. & de Bont E. S. Bone marrow-derived cells and tumor growth: contribution of bone marrow-derived cells to tumor micro-environments with special focus on mesenchymal stem cells. Crit Rev. Oncol. Hematol. 69, 187–198 (2009). 18675551 10.1016/j.critrevonc.2008.06.004

[b29] Siegel R., Ma J., Zou Z. & Jemal A. Cancer statistics, 2014. CA Cancer. J. Clin. 64, 9–29 (2014). 24399786 10.3322/caac.21208

[b30] Zhang X. *et al.* Exosomes in cancer: small particle, big player. J. Hematol. Oncol. 8, 83, doi: 10.1186/s13045-13015-10181-x (2015). 26156517 PMC4496882

[b31] O’Loughlin A. J., Woffindale C. A. & Wood M. J. A. Exosomes and the emerging field of exosome-based gene therapy. Curr. Gene Ther. 12, 262–274 (2012). 22856601 10.2174/156652312802083594

[b32] Chen T. S. *et al.* Mesenchymal stem cell secretes microparticles enriched in pre-microRNAs. Nucleic Acids Res. 38, 215–224 (2010). 19850715 10.1093/nar/gkp857 PMC2800221

[b33] Tan X., Gong Y.-Z., Wu P., Liao D.-F. & Zheng X.-L. Mesenchymal stem cell-derived microparticles: a promising therapeutic strategy. Int. J. Mol. Sci. 15, 14348–14363 (2014). 25196436 10.3390/ijms150814348 PMC4159854

[b34] Lee J.-K. *et al.* Exosomes derived from mesenchymal stem cells suppress angiogenesis by down-regulating VEGF expression in breast cancer cells. PloS one. 8, e84256, doi: 84210.81371/journal.pone.0084256 (2013). 24391924 10.1371/journal.pone.0084256 PMC3877259

[b35] Kogure T., Lin W. L., Yan I. K., Braconi C. & Patel T. Intercellular nanovesicle‐mediated microRNA transfer: A mechanism of environmental modulation of hepatocellular cancer cell growth. Hepatology. 54, 1237–1248 (2011). 21721029 10.1002/hep.24504 PMC3310362

[b36] Record M., Subra C., Silvente-Poirot S. & Poirot M. Exosomes as intercellular signalosomes and pharmacological effectors. Biochem. Pharmacol. 81, 1171–1182 (2011). 21371441 10.1016/j.bcp.2011.02.011

[b37] Théry C., Ostrowski M. & Segura E. Membrane vesicles as conveyors of immune responses. Nat. Rev. Immunol. 9, 581–593 (2009). 19498381 10.1038/nri2567

[b38] Sahoo S. *et al.* Exosomes from human CD34+ stem cells mediate their proangiogenic paracrine activity. Circ. Res. 109, 724–728 (2011). 21835908 10.1161/CIRCRESAHA.111.253286 PMC3201702

[b39] Kang K. *et al.* Exosomes secreted from CXCR4 overexpressing mesenchymal stem cells promote cardioprotection via Akt signaling pathway following myocardial infarction. Stem Cells. Int. 2015, 659890, doi: 659810.651155/652015/659890 (2015). 26074976 10.1155/2015/659890 PMC4436515

[b40] Takahashi Y. *et al.* Visualization and *in vivo* tracking of the exosomes of murine melanoma B16-BL6 cells in mice after intravenous injection. J. Biotechnol. 165, 77–84 (2013). 23562828 10.1016/j.jbiotec.2013.03.013

[b41] Lai C. P. *et al.* Dynamic biodistribution of extracellular vesicles *in vivo* using a multimodal imaging reporter. ACS Nano. 8, 483–494 (2014). 24383518 10.1021/nn404945r PMC3934350

[b42] Lim P., Patel S. A. & Rameshwar P. In Stem Cell-Based Tissue Repair (eds. Gorodetsky R. & Schäfer R.) Ch. 17, 346–365 (RSC Publishing) (2010).

[b43] Khakoo A. Y. *et al.* Human mesenchymal stem cells exert potent antitumorigenic effects in a model of Kaposi’s sarcoma. J. Exp. Med. 203, 1235–1247 (2006). 16636132 10.1084/jem.20051921 PMC2121206

[b44] Qiao L. *et al.* Suppression of tumorigenesis by human mesenchymal stem cells in a hepatoma model. Cell. Res. 18, 500–507 (2008). 18364678 10.1038/cr.2008.40

[b45] Cousin B. *et al.* Adult stromal cells derived from human adipose tissue provoke pancreatic cancer cell death both *in vitro* and *in vivo*. PloS one. 4, e6278, doi: 6210.1371/journal.pone.0006278 (2009). 19609435 10.1371/journal.pone.0006278 PMC2707007

[b46] Xu X. *et al.* Isolation and comparison of mesenchymal stem-like cells from human gastric cancer and adjacent non-cancerous tissues. J. Cancer Res. Clin. Oncol. 137, 495–504 (2011). 20473524 10.1007/s00432-010-0908-6 PMC11827778

[b47] Lin T.-M. *et al.* Isolation and identification of mesenchymal stem cells from human lipoma tissue. Biochem. Biophys. Res. Commun. 361, 883–889 (2007). 17679141 10.1016/j.bbrc.2007.07.116

[b48] Brune J. C. *et al.* Mesenchymal stromal cells from primary osteosarcoma are non-malignant and strikingly similar to their bone marrow counterparts. Int. J. Cancer. 129, 319–330 (2011). 20878957 10.1002/ijc.25697

[b49] Suzuki K. *et al.* Mesenchymal stromal cells promote tumor growth through the enhancement of neovascularization. Mol. Med. 17, 579–587 (2011). 21424106 10.2119/molmed.2010.00157 PMC3146617

[b50] Zhang T. *et al.* Bone marrow-derived mesenchymal stem cells promote growth and angiogenesis of breast and prostate tumors. Stem Cell. Res.Ther. 4, 70, doi: 10.1186/scrt1221 (2013). 23763837 PMC3707041

[b51] Hu G., Drescher K. M. & Chen X. Exosomal miRNAs: biological properties and therapeutic potential. Front. Genet. 3, 56, doi: 10.3389/fgene.2012.00056 (2012). 22529849 PMC3330238

[b52] Christianson H. C., Svensson K. J., van Kuppevelt T. H., Li J.-P. & Belting M. Cancer cell exosomes depend on cell-surface heparan sulfate proteoglycans for their internalization and functional activity. Proc. Natl. Acad. Sci. 110, 17380–17385 (2013). 24101524 10.1073/pnas.1304266110 PMC3808637

[b53] Chen T. S. *et al.* Enabling a robust scalable manufacturing process for therapeutic exosomes through oncogenic immortalization of human ESC-derived MSCs. J. Transl. Med. 9, 47, doi: 10.1186/1479-5876-1189-1147 (2011). 21513579 PMC3100248

[b54] Yeo R. W. Y. *et al.* Mesenchymal stem cell: an efficient mass producer of exosomes for drug delivery. Adv. Drug Deliv. Rev. 65, 336–341 (2013). 22780955 10.1016/j.addr.2012.07.001

[b55] Buyanovskaya O. *et al.* Spontaneous aneuploidy and clone formation in adipose tissue stem cells during different periods of culturing. Bull. Exp. Biol Med. 148, 109–112 (2009). 19902110 10.1007/s10517-009-0647-3

[b56] Zhou Y. F. *et al.* Spontaneous transformation of cultured mouse bone marrow–derived stromal cells. Cancer Res. 66, 10849–10854 (2006). 17108121 10.1158/0008-5472.CAN-06-2146

[b57] Chowdhury S. A. *et al.* Tumor-specificity and apoptosis-inducing activity of stilbenes and flavonoids. Anticancer. Res. 25, 2055–2063 (2005). 16158945

[b58] Senthilkumar K. *et al.* Quercetin regulates insulin like growth factor signaling and induces intrinsic and extrinsic pathway mediated apoptosis in androgen independent prostate cancer cells (PC-3). Mol. Cell. Biochem. 344, 173–184 (2010). 20658310 10.1007/s11010-010-0540-4

[b59] Igney F. H. & Krammer P. H. Death and anti-death: tumour resistance to apoptosis. Nat. Rev. Cancer. 2, 277–288 (2002). 12001989 10.1038/nrc776

[b60] Cao F. *et al.* *In vivo* visualization of embryonic stem cell survival, proliferation, and migration after cardiac delivery. Circulation. 113, 1005–1014 (2006). 16476845 10.1161/CIRCULATIONAHA.105.588954 PMC4701384

[b61] Kim J. E., Kalimuthu S. & Ahn B.-C. *In Vivo* Cell Tracking with Bioluminescence Imaging. Nucl. Med. Mol. Imag. 49, 3–10 (2015). 10.1007/s13139-014-0309-x PMC4354780 25774232

[b62] Keyaerts M., Caveliers V. & Lahoutte T. Bioluminescence imaging: looking beyond the light. Trends. Mol. Med. 18, 164–172 (2012). 22321645 10.1016/j.molmed.2012.01.005

[b63] Baker M. Whole-animal imaging: The whole picture. Nature. 463, 977–980 (2010). 20164931 10.1038/463977a

[b64] Zhao X. *et al.* Cysteine Dioxygenase Type 1 Inhibits Osteogenesis by Regulating Wnt Signaling in Primary Mouse Bone Marrow Stromal Cells. Sci.Rep. 6, 19296, doi: 19210.11038/srep19296 (2016). 26763277 10.1038/srep19296 PMC4725904

[b65] Imai T. *et al.* Macrophage-dependent clearance of systemically administered B16BL6-derived exosomes from the blood circulation in mice. J. Extracell. Vesicles. 4, 26238–26238 (2014). 25669322 10.3402/jev.v4.26238 PMC4323410

[b66] Lai C. P. *et al.* Visualization and tracking of tumour extracellular vesicle delivery and RNA translation using multiplexed reporters. Nat. Commun. 6, 7029, doi: 7010.1038/ncomms8029 (2015). 25967391 10.1038/ncomms8029 PMC4435621

