# Supplementary material for: In Vivo therapeutic potential of mesenchymal stem cell-derived extracellular vesicles with optical imaging reporter in tumor mice model
Source: Sci Rep. 2016 Jul 25;6:30418. doi: 10.1038/srep30418 (PMC4958922; doi:10.1038/srep30418)
Supplement: Supplementary Information [file srep30418-s1.pdf]

In Vivo therapeutic potential of mesenchymal stem cell-derived extracellular vesicles with optical imaging reporter in tumor mice model

Senthilkumar Kalimuthu[1], Prakash Gangadaran[1], Xiu Juan Li[1], Ji Min Oh[1], Ho Won Lee[1], Shin Young Jeong[1], Sang-Woo Lee[1], Jaetae Lee[1, 2], and Byeong-Cheol Ahn[1,*]

[1]Department of Nuclear Medicine, Kyungpook National University School of Medicine/Hospital, Daegu 700-721, Republic of Korea.

[2]Daegu-Gyeongbuk Medical Innovation Foundation (DGMIF), 80 Cheombok-ro, Dong-gu, Daegu 701-310, Republic of Korea.

* Corresponding author

Prof. Byeong-Cheol Ahn., M.D., Ph.D.,

Department of Nuclear Medicine,

Kyungpook National University School of Medicine and Hospital,

50, Samduk 2-ga, Jung Gu, Daegu-700-721, Republic of Korea.

Tel: 82-53-420-5583

Fax: 82-53-422-0864

Email: abc2000@knu.ac.kr

Supplementary Material:

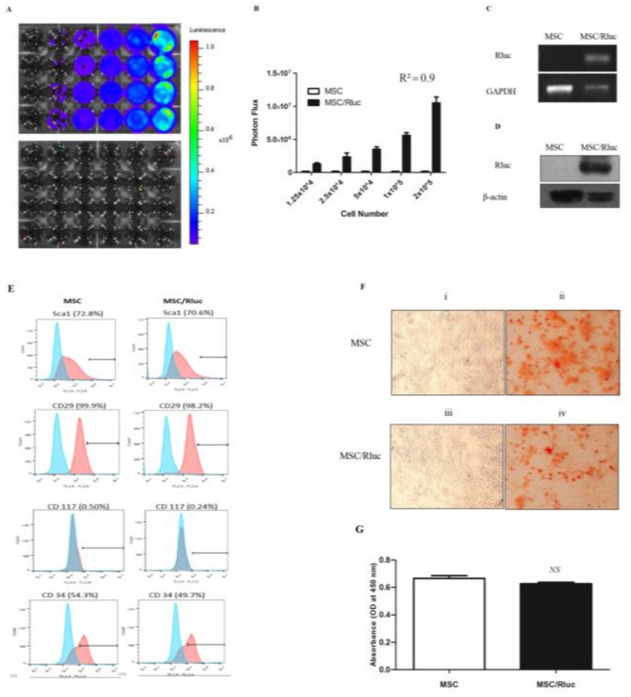

Supplementary Figure 1. Generation of stable reporter Rluc gene expression in mouse bone marrow-derived MSCs. (A) BLI of *in vitro* Renilla luciferase activity of MSC/Rluc and MSC. (B) Quantitative analysis of Rluc activity. (C) RT-PCR analysis of Rluc gene expression for MSC and MSC/Rluc cells. GAPDH was used as an internal control. (D) Western blot analysis of Rluc (37 kDa) protein in MSC and MSC/Rluc cells. β-actin was used for an internal control. Phenotype marker and cell proliferation analysis of MSC and MSC/Rluc cells. (E) Phenotype marker analysis for MSC and MSC/Rluc cells by flow cytometry. MSC and MSC/Rluc cells were positive for Sca-1 (Stromal cell antigen-1), CD-29, and CD-34, and negative for CD-117. (F) Osteogenic differentiation of MSC and MSC/Rluc cells determined by Alizarin Red S staining. Differentiation was induced with osteogenic medium for 14 days. (i) Control MSC medium (ii) Osteogenic medium-induced MSC (iii) Control MSC/Rluc cells (iv) Osteogenic medium-induced MSC/Rluc cells. Both MSC and MSC/Rluc cells indicated positive staining for Alizarin Red S whereas no staining was seen in undifferentiated control cells. (G) MSC and MSC/Rluc cell proliferation assay determined by CCK-8 analysis. MSC and MSC/Rluc proliferation rate was similar after 48 h. The data are expressed as the mean ± standard deviation (SD) of three independent experiments. *NS* denotes no significant changes.

A B

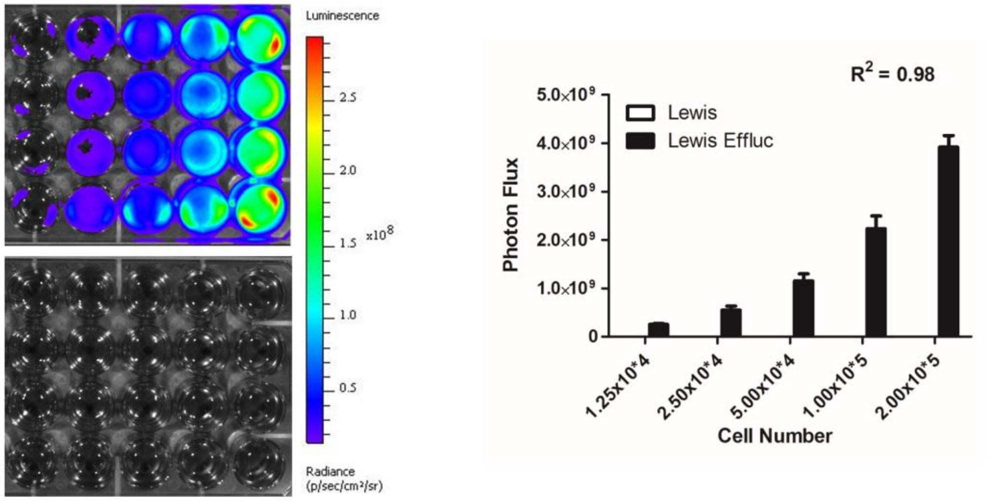

Supplementary Figure 2. Generation of stable reporter effluc expression in LLC cells. BLI of *in vitro* effluc activity of LLC and LLC-effluc cells. (B) Quantitative analysis of luciferase activity. Data are expressed as the mean ± standard deviation (SD) of three independent experiments.

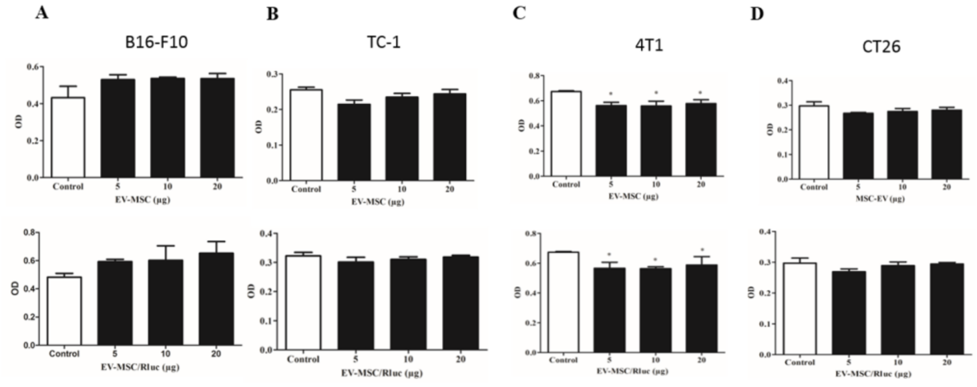

Supplementary Figure 3. Effect of parental EV-MSC and EV-MSC/Rluc on mouse cancer cell viability by CCK8 assay. (A) Melanoma cells, (B) uterine cervical cancer cells, (C) breast cancer cells, (D) colon cancer cells. Data are expressed as the mean ± standard deviation (SD) of three independent experiments, *$p < 0.05$, (by Student's *t*-test).

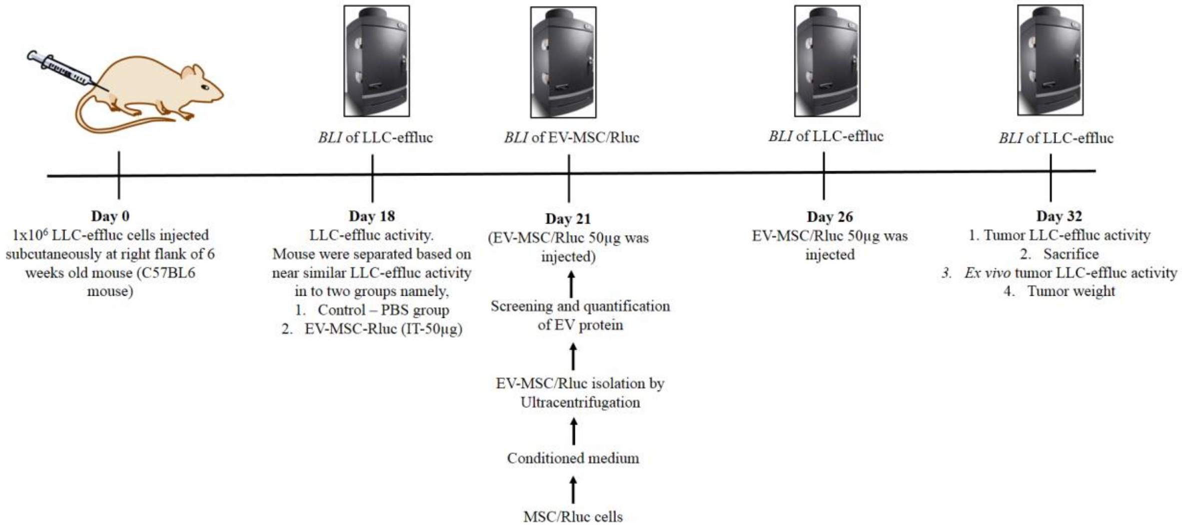

Supplementary figure 4. Schematic diagram of the *in vivo* experiment.

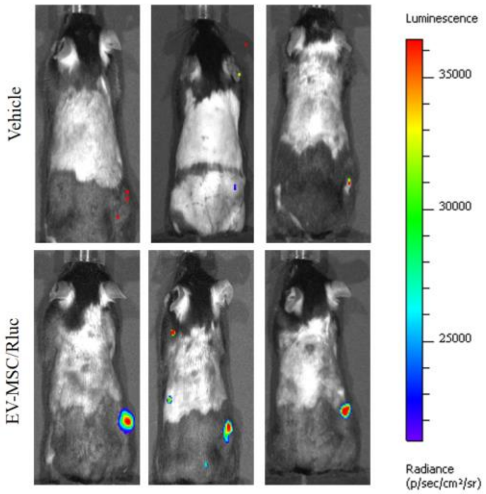

Supplementary Figure 5. *In vivo* imaging of intratumor (I.T.) administered EV-MSC/Rluc**.** Visualization of Rluc activity of LLC-effluc tumors I.T.-injected with EV-MSC/Rluc on day 21. Upper panel is control group treated with vehicle (PBS) alone. No appreciable signal was detected in PBS-injected mice. The lower panel shows tumors injected with 50 μg of EV-MSC/Rluc. Rluc activity was detected in EV-MSC/Rluc injected mice.
